# Increasing Complexity in Rule-Based Clinical Decision Support: The Symptom Assessment and Management Intervention

**DOI:** 10.2196/medinform.5728

**Published:** 2016-11-08

**Authors:** David F Lobach, Ellis B Johns, Barbara Halpenny, Toni-Ann Saunders, Jane Brzozowski, Guilherme Del Fiol, Donna L Berry, Ilana M Braun, Kathleen Finn, Joanne Wolfe, Janet L Abrahm, Mary E Cooley

**Affiliations:** ^1^ School of Medicine Department of Community & Family Medicine Duke University Durham, NC United States; ^2^ Klesis Healthcare Durham, NC United States; ^3^ Family Medicine of Albemarle Charlottesville, VA United States; ^4^ Medengineers Informatics Charlottesville, VA United States; ^5^ Dana-Farber Cancer Institute The Phyllis F. Cantor Center Boston, MA United States; ^6^ Independent Clinical Informatics Consultant Boston, MA United States; ^7^ Department of Biomedical Informatics University of Utah Salt Lake City, UT United States; ^8^ Department of Psychosocial Oncology and Palliative Care Dana-Farber Cancer Institute Boston, MA United States; ^9^ City of Hope Clinical Trials Office Duarte, CA United States

**Keywords:** rule-based clinical decision support, clinical algorithms, Web services, software as a service, symptom management, patient-reported outcomes, lung cancer

## Abstract

**Background:**

Management of uncontrolled symptoms is an important component of quality cancer care. Clinical guidelines are available for optimal symptom management, but are not often integrated into the front lines of care. The use of clinical decision support (CDS) at the point-of-care is an innovative way to incorporate guideline-based symptom management into routine cancer care.

**Objective:**

The objective of this study was to develop and evaluate a rule-based CDS system to enable management of multiple symptoms in lung cancer patients at the point-of-care.

**Methods:**

This study was conducted in three phases involving a formative evaluation, a system evaluation, and a contextual evaluation of clinical use. In Phase 1, we conducted iterative usability testing of user interface prototypes with patients and health care providers (HCPs) in two thoracic oncology clinics. In Phase 2, we programmed complex algorithms derived from clinical practice guidelines into a rules engine that used Web services to communicate with the end-user application. Unit testing of algorithms was conducted using a stack-traversal tree-spanning methodology to identify all possible permutations of pathways through each algorithm, to validate accuracy. In Phase 3, we evaluated clinical use of the system among patients and HCPs in the two clinics via observations, structured interviews, and questionnaires.

**Results:**

In Phase 1, 13 patients and 5 HCPs engaged in two rounds of formative testing, and suggested improvements leading to revisions until overall usability scores met a priori benchmarks. In Phase 2, symptom management algorithms contained between 29 and 1425 decision nodes, resulting in 19 to 3194 unique pathways per algorithm. Unit testing required 240 person-hours, and integration testing required 40 person-hours. In Phase 3, both patients and HCPs found the system usable and acceptable, and offered suggestions for improvements.

**Conclusions:**

A rule-based CDS system for complex symptom management was systematically developed and tested. The complexity of the algorithms required extensive development and innovative testing. The Web service-based approach allowed remote access to CDS knowledge, and could enable scaling and sharing of this knowledge to accelerate availability, and reduce duplication of effort. Patients and HCPs found the system to be usable and useful.

## Introduction

Clinical decision support (CDS) derived from clinical algorithms (ie, rule-based) is essential for improving the quality and safety of health care [1]. In spite of the critical nature of this resource, much of rule-based CDS to date has been relatively simplistic, and few examples of complex decision algorithms with dozens of decision points have been implemented [2,3]. As increasingly complex clinical protocols are implemented through CDS, innovative approaches will be required to thoroughly and rigorously validate the accuracy of these CDS systems [4].

In order to fulfill the clinical expectations of CDS in the future, the next generation of rule-based CDS will need to mature to: (1) accommodate increasing clinical complexity; (2) respond to current patient status by incorporating real-time clinical information, including patient-reported data; and (3) increase efficiency by allowing for scaling and portability through reuse of decision logic by separating the end user application from the decision engine. In this project, we developed a CDS system that supported all three of these features. This system supported the complex challenge of simultaneously managing multiple symptoms (anxiety, depression, dyspnea, fatigue, and pain) in patients with lung cancer, the collection of real-time symptom data from patients, and potential reutilization of algorithm knowledge via Web services.

Symptom management in lung cancer patients is complex, and uncontrolled symptoms have been associated with increased emotional distress, decreased health-related quality of life, and even decreased survival [5-9]. The majority of lung cancer patients have high levels of disease-related symptomatology, as well as psychological distress at presentation [10-14]. Optimal management requires attention to multiple symptoms. To date, the majority of studies aiming to enhance symptom management have addressed the treatment of individual symptoms [15-19]. New approaches to manage multiple distressing symptoms are needed. National groups have called for improving symptom management and palliative care across the cancer continuum, and for supporting improved quality of care with the use of health care information technology [20-22]. In a prior project, we convened multidisciplinary panels of clinical experts to develop computable symptom management algorithms for multiple symptoms based on national clinical practice guidelines [23]. These algorithms provided recommendations for specific pharmacological and behavioral interventions–tailored to a patient’s age, comorbidities, laboratory values, current medications, and patient-reported symptom severity–to manage anxiety, depression, dyspnea, fatigue, and pain. The complex algorithms, and their integration with one another, approximated the cognitive processes of clinical experts and considered multiple factors that may aggravate and/or alleviate common cancer symptoms. Further information regarding the expert panel and processes used to develop the computable algorithms has been published previously [23].

In this paper we report on the development, testing, and contextual evaluation of the Symptom Assessment and Management Intervention for Lung cancer (SAMI-L) CDS system that was based on these algorithms, in two hospital-based clinics. In *Phase 1*, our objective was to develop usable and acceptable user interfaces to accurately capture the patient-reported and clinical data required to process the algorithms, and to display guideline-based recommendations in interpretable and actionable ways to health care providers (HCPs). In *Phase 2*, our objective was to program and test the accuracy of the algorithms and the integrated system. In *Phase 3*, our objective was to evaluate the use of the system by patients and HCPs in the clinical setting.

### System Description

**Figure 1:**
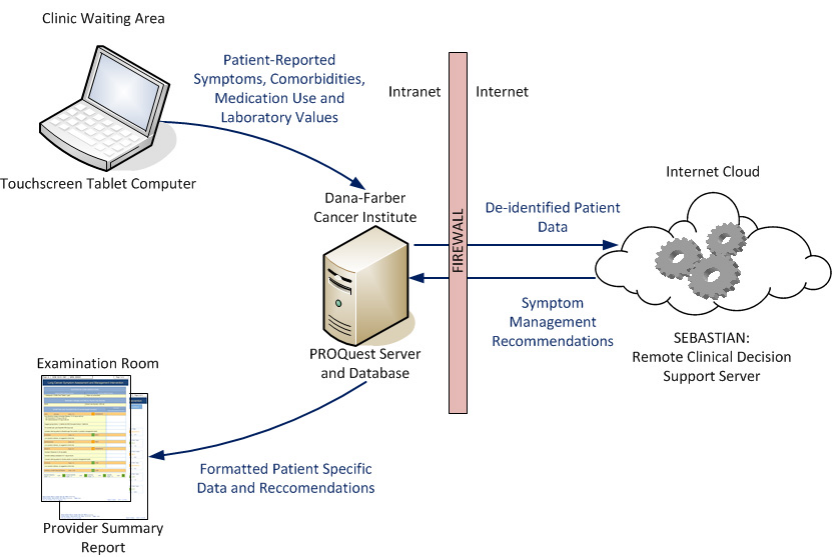
SAMI-L system architecture and overview.

The SAMI-L system consists of three components: (1) a Web-based assessment tool for collecting patient-reported data on symptom severity, medications, and laboratory values using a touch screen notebook computer. This tool uses standardized patient-reported outcome questionnaires that have been used previously with cancer patients, and are among the most commonly used measures in such studies [24-27]; (2) a *decision engine* known as the System for Evidence-Based Advice through Simultaneous Transaction with an Intelligent Agent Across a Network (SEBASTIAN) [28], accessed remotely using Web services; and (3) printed reports for clinicians that summarize patient data and present patient-specific recommendations (Figure 1).

Figure 1 identifies the components of the SAMI-L system and the data flow between these components. Patients and research assistants entered data on a touch screen notebook computer in the clinic waiting area. These data were then transmitted, with a session identification number and no personal health information, through the PROQuest server to the SEBASTIAN decision support engine using Web services. After processing the data, the recommendations were returned from the decision engine through Web services to the PROQuest server where they were formatted into patient reports. These reports were then printed and delivered to the healthcare provider in the examination room.

The *decision engine* was built using a Web service-based CDS tool known as SEBASTIAN [28]. The SEBASTIAN system is one of the initial decision engines that implemented CDS using Web services [29]. This system provided the foundation for the evolving HL7 Decision Support Service standard and has been described previously [28].

SEBASTIAN receives data from remote client applications structured in a common *language* known as eXtensible Markup Language (XML). Using this Web service framework, decision logic can be centralized in SEBASTIAN for use by many systems at different sites, thus enabling the sharing of computable knowledge across multiple remote locations [30]. The complex symptom management algorithms were represented in the form of procedural rules, and implemented into SEBASTIAN using an object-oriented computer programming language (Java). In order to generate specific symptom management care recommendations, the symptoms, medications, and laboratory values were submitted as Web service requests from a server in Boston, Massachusetts to a cloud-based server for processing by the SEBASTIAN inference engine [31]. Submitted patient information was distinguished by a unique session identifier so that only nonidentifying patient information was transmitted to the CDS server. Complete traversal of each decision node was critical for generating correct recommendations, so we programmed the SAMI-L system to function only if all required data were available. Accordingly, each clinical rule would determine that all of the required data were present before running. If data were missing, the system would send a message stating that the available data were insufficient to run the algorithm.

**Figure 2:**
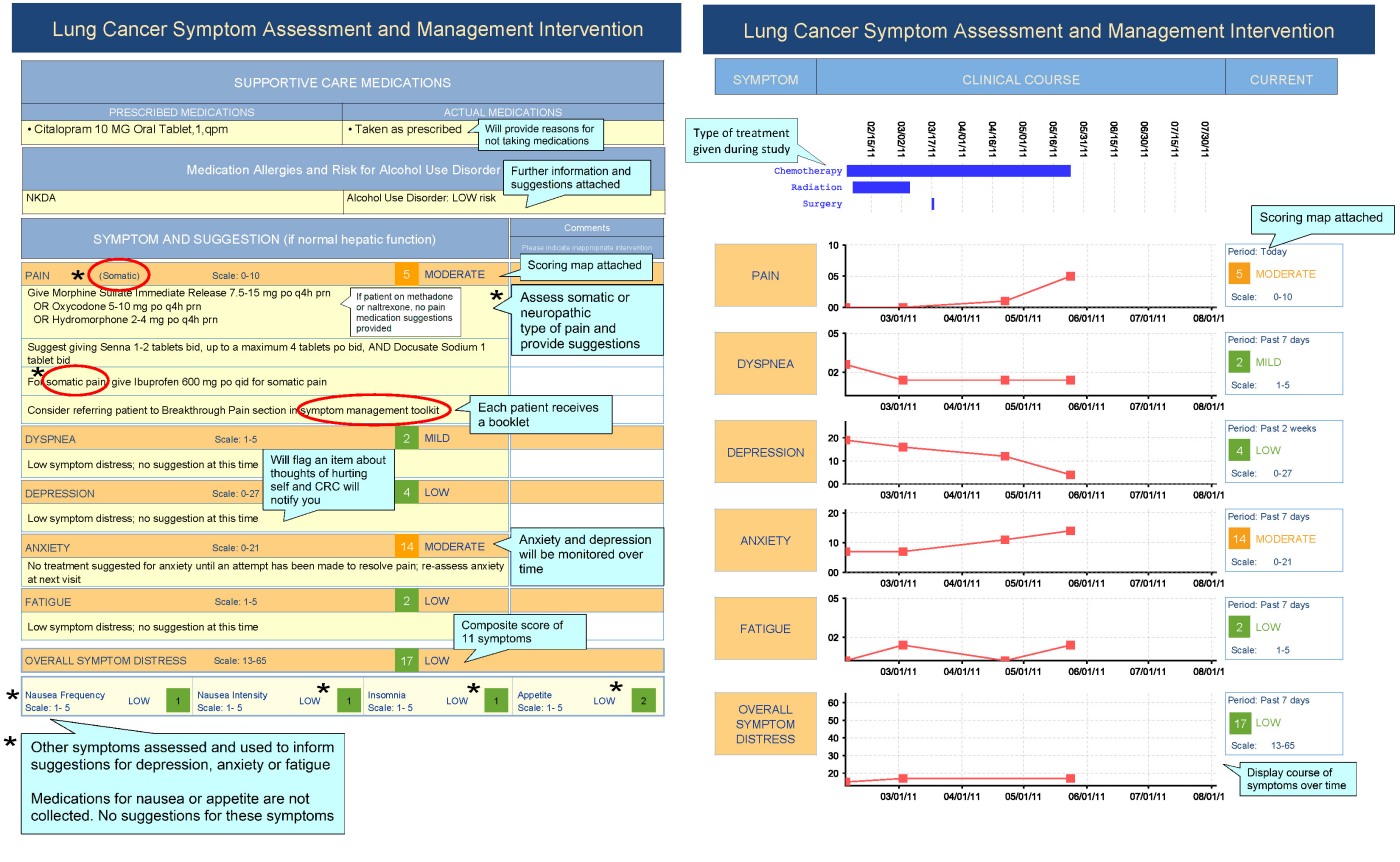
Sample report produced by SAMI-L.

SAMI-L also generated a printed report for clinicians to use during the clinical visit (Figure 2). This two-page report included a summary of patient-reported data along with patient-specific recommendations based on the symptom management algorithms. This information was presented in lists, tables, charts with color coding, and trend graphs to make it easily consumable by clinicians. The symptom management guidance was based on the severity of a patient’s symptoms. Guidance included specific suggestions for use of medications (including recommendations to initiate medications or explicit adjustments for medication doses), laboratory tests, supportive care referrals (ie, social work, palliative care, psychiatry), and use of a self-care symptom management toolkit for patients that provided behavioral self-care suggestions [32,33].

The left panel of Figure 2 provides the data from which the care recommendations were derived, including the current medications, medication allergies, alcohol use history, and the patient-reported level of distress by individual symptoms. Level of patient symptom distress was color-coded with green, yellow, and red to indicate increasing levels of distress. Explicit, patient-specific care guidance recommendations are provided with each individual symptom, as determined from the care algorithm. The right panel of Figure 2 shows a time course summary of a patient’s treatments, and a cumulative graphical summary of changes in a patient’s levels of symptom distress over time by each individual symptom. Figure 2 first appeared in Cooley et al [34].

## Methods

### Phase 1

In *Phase 1,* we conducted iterative usability testing of user interface prototypes with patients and HCPs in two thoracic oncology clinics. The expert panels had created computable symptom management algorithms that specified validated patient-reported symptom measures and clinical data that were required to process the algorithms in a previous project [23]. To create the patient component of the system, we constructed validated self-report symptom assessment questionnaires measuring the targeted symptoms, and data entry interfaces for required medication history and clinical variables. The questionnaires were constructed using an existing Web-based data collection platform at Dana-Farber Cancer Institute (DFCI). Patient participants were >21 years of age, English speaking, diagnosed with Stage III or IV nonsmall cell lung cancer, had limited or extensive stage small cell lung cancer or new recurrence of disease, were receiving care in the outpatient setting, and were actively receiving cancer-directed treatment. These patients were recruited for 60-minute usability test sessions. We oversampled patients from Boston Medical Center (BMC), a community-based safety-net hospital, to ensure representation of patient users with lower literacy and lower familiarity with computers. A usability interviewer from the Dana-Farber/Harvard Cancer Center Health Communication Core (HCC) used a structured interview guide to observe and elicit feedback on understanding of the assessment, ease of navigation, helpfulness of the program, the amount of time required to complete the program, and overall user satisfaction. The interviewer also observed mock reviews of medication history by the study coordinator, as would be required to obtain data needed to process the algorithms. Patient participants completed the Acceptability E-Scale [35] and a demographic questionnaire at the end of the session. Following the appropriate protocol, two or more rounds of testing were required until acceptability scores met the predefined threshold of an average score of 4 on a 5-point scale (1=low; 5=high) for each item, or a composite score of >24 across six items.

To create the HCP component of the system, prototype graphical summary reports of the CDS recommendations for symptom management were developed by a graphic designer in the HCC. We recruited eligible HCPs, who were attending physicians or nurse practitioners in the two thoracic medical oncology clinics, and randomized them to intervention or usual care arms for the trial. Participants in the intervention arm were invited to participate in formative usability testing of the reports. We conducted 30-minute usability sessions in which HCPs were presented with high fidelity mock reports of patients’ current and historical symptom status, and recommended pharmacologic and behavioral interventions. A research team member followed a structured script to solicit feedback and probe understanding of layout, content, and visual style of each section of the report. Participants then completed standard usability rating questionnaires [36,37] and a demographic questionnaire. Following the appropriate protocol, two or more rounds of testing were required until acceptability scores met the predefined threshold of an average of 4 on 5-point scale (1=low; 5=high) across all items.

### Phase 2

In *Phase 2,* we programmed the five complex algorithms (anxiety, depression, dyspnea, fatigue, and pain), which were derived from clinical practice guidelines, into a rules engine that used Web services to communicate with the end-user application. We conducted unit testing of algorithms using a stack-traversal tree-spanning (STTS) methodology to identify all possible permutations of pathways through each algorithm, to validate accuracy. The symptom management algorithms defined by the expert panels required >30 unique data elements (Multimedia Appendix 1) and were developed to address multiple clinical issues for appropriate symptom management (Multimedia Appendix 2).

Multimedia Appendix 1 provides information about the data requirements that were needed to inform the algorithms to generate specific recommendations for symptom management, the standardized assessment instruments that were used to collect the data, and the source of the data collection. Multimedia Appendix 2 provides information about the type of recommendations that were provided for each of the five algorithms (anxiety, depression, dyspnea, fatigue, and pain) and the specific data elements that were required to generate those recommendations.

### Algorithm Complexity

In order to quantify the complexity of the symptom management algorithms, we determined the number of decision nodes and unique pathways within each algorithm. For this purpose, we counted a *decision node* as a point within an algorithm where the logic could branch in two or more directions. In some algorithms, specific clinical parameters (ie, renal function) appeared in two or more distinct parts of the algorithm, based on when in the course of decision-making kidney function should be considered. In such cases, each instance of the renal function node would be added to the total node count for the algorithm. A *pathway* was defined as a unique sequence of branches through the algorithm that began at the entry point of the algorithm and ended at a specific end node from which no additional decision nodes followed.

After programming logic content into the SEBASTIAN decision engine, the number of decision nodes in the symptom management algorithms ranged from a low of 29 in the fatigue algorithm to a high of 1425 in the pain algorithm (Table 1). Traversal of these algorithms across all possible variable permutations identified a low of 19 unique pathways in the fatigue algorithm and a high of 3194 pathways in the pain algorithm (Table 1).

**Figure 3:**
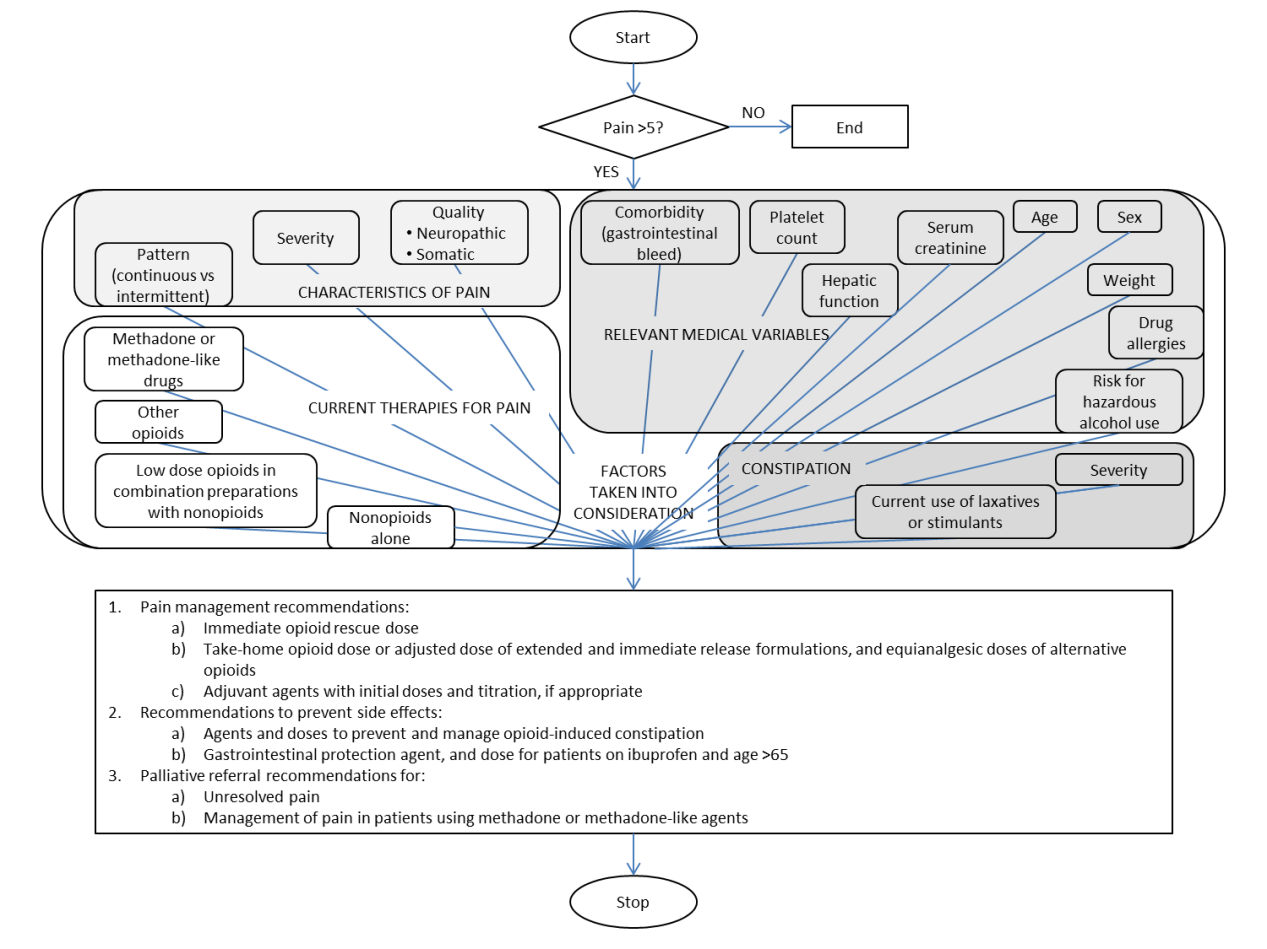
Pain algorithm data components and recommendations.

As an illustration of the complexity of the algorithm for pain management, the diagram in Figure 3 portrays the factors that were considered in generating recommendations for care guidance, along with the types of recommendations that are typically considered for patients experiencing significant levels of pain. Figure 3 displays a schematic representation of the pain algorithm to demonstrate the complexity of the logic considered for managing pain. The upper component of Figure 3 illustrates the multiple factors that were taken into consideration, in order to generate care guidance recommendations to manage pain. Factors include the characteristics of the pain, the current therapy for the pain, relevant medical variables, and issues related to opioid-induced constipation. The lower component of Figure 3 summarizes the types of recommendations that were produced, including recommendations for pain management, recommendations to prevent side effects from pain medications, and recommendations for palliative care referrals.

**Table 1:** Number of unique pathways and decision nodes in symptom management algorithms.

Rule	Decision Nodes	Separate Pathways
Anxiety	45	43
Depression	42	39
Fatigue	29	19
Pain	1425	3194
Dyspnea	87	113

### System Testing Approach

The complex care algorithms developed to address simultaneous symptom management required new methods to thoroughly and rigorously validate the accuracy of the CDS recommendations. Accordingly, a systematic approach was developed to ensure that all of the possible permutations arising from hundreds of branching pathways had been assessed. First, in order to identify errors during unit testing, the study team selected hundreds of representative instances of automatically generated test cases with predetermined recommendations. Next, the test cases were submitted to SEBASTIAN and mismatches between the newly-generated recommendations and the expected recommendations were identified. The advantage of this approach was that future changes in algorithms could be tested by running the same test cases. Results of hundreds of test cases were also manually compared to the algorithm flowcharts (approved by an expert clinician) to ensure that there were no logic errors in the algorithms.

Second, in order to identify errors during integration testing, the study team developed a set of 10 test cases. These test cases were sent to the CDS Web service from the study sites, using the data collected via SAMI-L. The recommendations generated from SEBASTIAN were reviewed by a clinical expert to ensure their accuracy. In addition, the display of patient data, and the resulting recommendations that were part of the HCP report, were verified to ensure accuracy.

Finally, we created a systematic and reusable testing approach to validate the accuracy of complex care protocols using an STTS algorithm. For each algorithm, using an XML text editor, we created an XML data input file with data parameters targeting boundary conditions for each decision node. All possible permutations for traversing all of the pathways through each protocol were created using an XML-based STTS algorithm written in Java. The data elements defining each permutation were sequentially submitted as Web service requests to the decision engine. Each resultant set of recommendations was paired with the data set used to generate the response, and Altova MapForce [38] auto-generated Java code was used to map the input and output parameters to a queryable relational database. Initially, research staff rigorously queried the database to confirm that the correct recommendations had been generated from each paired variable-input-recommendation-output data set. Based on these systematic queries, inconsistencies in the logic were identified. The development team then corrected the logical inconsistencies by modifying the flow diagram and associated algorithms to correct the erroneous logic. The testing cycle was then repeated to ensure accuracy of the decision logic. In final testing, we automated the validation of the data-recommendation pairs using a unit testing approach with a set of manually validated test cases serving as the standard (ie, if new rule input-output parameters did not correspond with input-output parameters from a validated database, then new rule logic errors would be addressed). Care recommendations provided guidance that could directly impact patient care, so we required 100% accuracy of the generated recommendations (in terms of agreement with the stipulated algorithm) before the CDS for management of each symptom was moved to production. A clinical expert (JLA) reviewed all recommendations generated by the algorithms to ensure accuracy.

**Figure 4:**
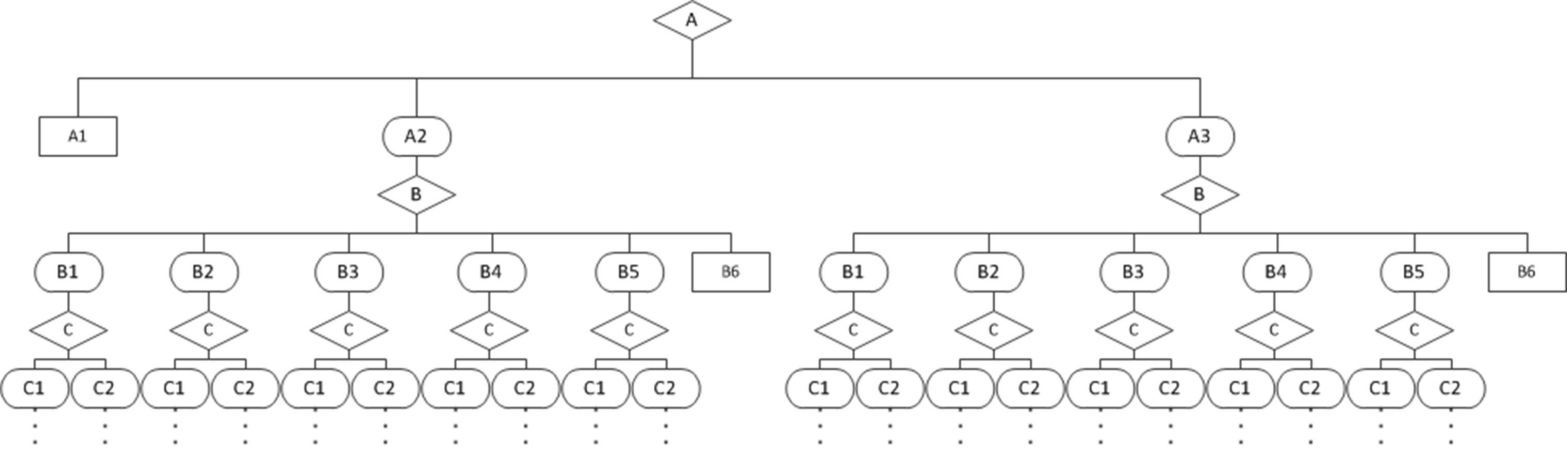
Sample of stack-traversal tree-spanning algorithm approach.

As an illustration of the STSS algorithm approach, a subsection of the pain management algorithm is shown in Figure 4. The diagram in Figure 4 illustrates three levels of decision nodes from the pain algorithm. The first level addresses patient-reported pain severity, which is categorized into three groups. The second level represents a patient’s opioid use within the past 24 hours, which is categorized into six groups. The third level depicts a patient’s creatinine clearance, which is categorized into two groups.

The STTS algorithm would follow every pathway to an end node while keeping a record of branches that had not yet been traversed (ie, the stack). After processing an end node (ie, a unique clinical decision pathway), the algorithm would then revisit the last node it had placed on the stack (ie, *pop* it off the stack) and then attempt to use this *popped* node's connections to find a new unique end node that had not yet been processed. Through this systematic traversal of the clinical algorithm, every possible pathway was identified and sample patient variables were set to specific values to ensure that each path would be traversed with every testing cycle. Since the *correct* recommendations that should result from every pathway were defined, and since every input data set could be paired with the anticipated output recommendations, comparison of the actual output recommendations with the expected recommendations led to the identification of errors in the algorithm logic. In the pain algorithm case, if the input variables were set with a pain score of 8 (node A3 in Figure 4), with an opioid use history of slow release opioids only (node B4), and with a normal kidney function (node C1), the algorithm should generate a recommendation to add immediate release opioids to the patient’s pain control regimen.

### Phase 3

In *Phase 3,* we evaluated clinical use of the system among patients and HCPs in the two clinics via observations, structured interviews, and questionnaires. Patients and HCPs meeting the same criteria employed in *Phase 1* were recruited to participate in a feasibility trial (details previously reported [34]). In the final six months of the trial, we conducted a user evaluation. Data were collected from patients and HCPs using observations of CDS system use, standardized questionnaires, and structured interviews. Descriptive statistics were used to analyze quantitative data on the Acceptability E-Scale and scoring with preset item threshold of 4 on the 1-5 response scale. Qualitative data were content analyzed using NVivo 9.0 software [39].

## Results

### Phase 1

13 patients participated in two rounds of testing **:** four were from DFCI and nine were from BMC. The sample was 62% (8/13) female, 82% (9/11; 2 missing) had less than a high school education, 54% (7/13) reported minority race, 46% (6/13) reported that they never or rarely used computers, and median age was 57 years. The usability testing scores for all patients in both rounds exceeded minimal acceptability scores. The mean scores for all items of the Acceptability E-Scale were >4 and composite acceptability scores averaged 27.5 for *round 1* and 26.9 for *round 2*, which exceeded the predefined threshold of 24. Based on patient comments, design features were tailored to accommodate computer use in older adults who were acutely ill, assist low literacy adults with no previous use of computers, ensure understanding of the time anchors, and enable accurate data collection regarding self-report of symptoms and medication use.

Five HCPs participated in two rounds of usability sessions: four were from DFCI and one was from BMC. The sample was 80% (4/5) male, 60% (3/5) white, and median age was 49 years. The average usability testing subscale scores for participants ranged from 3.3 to 3.9 in *round 1* and 3.6 to 3.9 in *round 2*. Based on HCP comments, design features were tailored to ensure that summary reports were easy to read in a busy clinical setting, confirm that no extra work was required to access the forms, ensure that decision support was timely so that it could be used during the clinical visit, and guarantee that all of the symptoms that were assessed using the algorithms were displayed and easily seen by the HCPs.

### Phase 2 - Testing Results

Unit testing required an estimated 240 person-hours over nine months, including time between rounds for corrections. The simplest algorithm (fatigue) required the least testing: four rounds over eight weeks. The most complex algorithm (pain) included the adjustment and conversion of opioid doses, recommendations of specific doses of medications for neuropathic and somatic pain, and addition of bowel regimens. The pain algorithm required five rounds of testing over six months. The results of unit testing identified both runtime and logical programming errors prior to clinical application. Examples of logical errors discovered during unit testing included morphine equivalent dosing irregularities and flow chart/algorithm wording that necessitated clarification for correct representation in programming logic. A small number of problems related to incomplete reasoning or inconsistent recommendations of the original algorithms were also identified, such as the potentially confusing simultaneous recommendations to increase a medication for depression but maintain the medication for anxiety. Clinical experts defined solutions in these cases. After each revision to an algorithm and programmed rule, each algorithm was tested again to ensure adequacy of the revision until no further errors were identified.

Integration testing was conducted in twelve rounds over eight weeks, using 10 test cases, and required an estimated 40 person-hours. Most identified errors were due to incorrect submission of data from the clinical site, such as an *unevaluable date* format. Integration testing also identified errors in display of data on the clinician report, and was used to define final requirements for the report. Each error was addressed and tested in the subsequent round until no further errors were identified. At the end of the testing, the rules were 100% accurate once all errors were corrected.

Using the STTS method described above, we generated all possible combinations of data parameters and variable values to enable validation of the five complex symptom management algorithms. Two illustrative sets of paired data input parameters, and their corresponding recommendation outputs, are shown in Table 2.

### Phase 3

43 patients (100% of those invited) participated in the evaluation: 42 were from DFCI and one was from BMC. The sample was 58% (25/43) female, and 95% (40/43; 1 missing) white, had a median age of 60, with 70% (30/43) reporting some college education, and 72% (31/43) reporting using computers often or very often. Participants completed the symptom self-report in an average of seven minutes, with the most common technical problem being timing out from the waiting room wireless connection, while medication review took less than two minutes on average. Average acceptability item scores for SAMI-L ranged from 4.21 to 4.98 (on a 1-5 scale). The average total score for the acceptability scale was 28 (of 30), exceeding the predefined threshold of 24 for acceptability. Most patients (58%, 25/43) would prefer assessments at every clinic visit, versus a greater or lesser frequency (16%, 7/43) or no preference (26%, 11/43). The majority of participants (72%, 31/43) preferred completing assessments during clinic visits, versus at home (12%, 5/43) or no preference (16%, 7/43), because it gave them something to do while waiting and was a more reliable way to ensure completion of the report. Facilitators for use included: improved communication with providers, having time to reflect on symptoms before the visit, helping pinpoint problems, and ease of use. The main barrier to use was unclear or limited options on SAMI-L questionnaires. Patients suggested having open-ended questions to identify additional issues of concern.

13 of 14 (93%) HCP participants randomized to the intervention arm participated in the evaluation: 11 HCPs were observed in 42 instances of receiving a SAMI-L report, and 13 HCPs completed structured interviews and usability questionnaires. HCP participants included seven physicians and six nurse practitioners. The sample was 54% (7/13) male, with median age of 40, and had a median of 12 years of experience in oncology. In 79% (33/42) of observations, HCPs received the report on average 21 minutes before the visit and took <1 minute to review the report. Usability scores for the report ranged from an average of 3.2 for usefulness to 4.5 for organization (on a 1-5 scale). Two-thirds of HCPs (9/13) reported using the algorithm-derived recommendations for pain most often, and those for dyspnea the least. Management of dyspnea was perceived as complex, and algorithm suggestions were seen as being too generic. Another barrier identified was lack of integration of the report into the flow of care. Facilitators of use were the reports’ colorful scales and line graphs used for tracking symptoms. Calculations for opioid dosing, identification of patient distress, and suggestions for managing fatigue and opioid-induced constipation were perceived as helpful.

**Table 2:** Pairing of input data parameters with resultant recommendations

Input Data Parameters	Resultant Recommendations
• Pain self-report=6 (moderate) • Intermittent pain • Pain is achy, sharp, or in one spot • No current opioid medications • Serum creatinine=0.9 • Sex=male • Age=67 • Weight=84 kg • Platelets=183,000/mL • No history of gastrointestinal bleed • Patient-Reported Outcomes version of the Common Terminology Criteria for Adverse Events bowel score=0 (no constipation) • No current bowel medications	• Give morphine sulfate Immediate Release 7.5-15 mg by mouth every 4 hours as needed • OR oxycodone 5-10 mg by mouth every 4 hours as needed • OR hydromorphone 2-4 mg by mouth every 4 hours as needed
	• Give acetaminophen 1000 mg by mouth three times a day for somatic pain NOT to exceed 3000 mg per day • OR ibuprofen 400 mg by mouth three times a day for somatic pain with omeprazole or pantoprazole 20 mg by mouth daily for GI protection
	• Suggest giving senna 1-2 tablets twice a day, up to a maximum of 4 tablets twice a day, AND docusate sodium 1 tablet twice a day, for prevention or treatment of opioid-induced constipation
• Pain self-report=8 (severe) • Constant pain • Pain is burning or shooting • Current opioid dose prescribed: oxycodone immediate release 15 mg by mouth, every 4 hours as needed, and oxycodone extended release 60 mg, by mouth twice a day, with actual past 24-hour oxycodone use equal to the maximum dose of 90 mg immediate release and 120 mg extended release • Serum creatinine=2.3 • Sex=female • Age=53 • Weight=61 kg • Patient-Reported Outcomes version of the Common Terminology Criteria for Adverse Events bowel score=3 (moderate, Grade 2 constipation) • Taking sennosides, United States Pharmacopeia 17 mg, 2 tablets, twice a day	• For symptom relief, give oxycodone at 30 mg by mouth. If pain >7 after 1 hour, suggest a palliative care consult. If pain is <6 after 1 hour, suggest you use one of the following combinations of sustained release and rescue dose opioids. Adjust to available formulations. • If oxycodone sustained release preferred: ○ Give oxycodone sustained release 120 mg by mouth twice a day, OR 80 mg by mouth three times a day ○ Give oxycodone immediate release 30 mg by mouth every 4 hours as needed • If transdermal fentanyl patch preferred: ○ Give transdermal patch 175 mcg/hr ○ Give oxycodone immediate release 30 mg by mouth every 4 hours as needed OR hydromorphone immediate release 15 mg by mouth every 4 hours as needed.
	• Suggest giving gabapentin 100 mg by mouth twice a day from days 1-7, then 200 mg by mouth twice a day from days 8-28, for neuropathic pain • If ineffective after 28 days: discontinue gabapentin and give pregabalin 50 mg by mouth twice a day from days 1-7, increasing to 75-100 mg by mouth twice a day from days 8-28. • If pregabalin ineffective after 28 days, call palliative care consult
	• Suggest titrating current 2.0 sennosides tablets by mouth twice a day, up to a maximum 4 tablets by mouth twice a day, to reach goal bowel function of either 1 bowel movement per day or 1 bowel movement every other day
	• AND give milk of magnesia 30mL once daily OR dulcolax 10 mg by mouth or by rectum once daily OR miralax 17 g once daily

## Discussion

In this paper we described the development and testing of a CDS system, the SAMI-L, that used complex algorithms to address the simultaneous management of five distressing symptoms in lung cancer patients. In previous studies, CDS was used to identify the presence of a single symptom using an algorithm with less than a dozen decision nodes that generated general recommendations [12,22], whereas the algorithms that were developed and tested in this project focused on five symptoms that contained decision nodes that varied from 29 for fatigue to 1425 for pain. Thus, the algorithms developed for this study were complex due to the number of symptoms addressed, and the number of decision nodes was large compared to previous studies. The complexity of these algorithms required a novel and rigorous approach to testing. The CDS system was acceptable and useful for patients and HCPs in preclinical and clinical settings.

The successful deployment of SAMI-L advances the field by demonstrating that complex clinical algorithms can be invoked in rule-based CDS systems to generate detailed patient-specific recommendations for use in the management of multiple symptoms at the point-of-care using patient-entered data. Most previously reported rule-based CDS systems have contained fewer than a dozen decision nodes and required only a small number of data parameters to function [40-42].

While SAMI-L provides an example for increasing the logic complexity of rule-based CDS systems, we recognize that SAMI-L represents only one approach to CDS (ie, CDS driven by explicit care algorithms) and that other approaches exist for CDS that manage even greater levels of complexity. Perhaps the most complex CDS tool described to date is the Watson technology developed by IBM [43]. In contrast to the defined rules of SAMI-L, Watson uses sophisticated natural language processing, and powerful information mining and retrieval capabilities to provide clinical guidance [44]. Watson-enabled CDS may reflect one future direction for CDS; however, we maintain that there is still a role for CDS systems that facilitate adherence to defined evidence-based best practices, as shown with SAMI-L. Rule-based CDS can be built with currently available technology in areas for which guidelines are available. As long as boundaries are clearly defined (eg, normal renal function in SAMI-L), rule-based CDS can be robust and promote guideline adherence.

In addition to the high-powered information mining and retrieval CDS approach enabled by Watson, another approach to enable complex CDS includes supervised learning models. While these approaches are able to support complex decisions, they require large sets of labeled data for algorithm training, often lack generalizability, are difficult to ensure replicability, and are not always able to provide the rationale for CDS recommendations.

Within the domain of CDS for symptom management, SAMI-L advances the field by supporting simultaneous management of multiple distressing symptoms in patients with lung cancer, in contrast to most previously reported systems that focus on a single symptom or problem [15,16]. The SAMI-L system also incorporates a measurement-based approach using patient-reported symptom severity, age, comorbidities, laboratory values, and adherence to medications to instantiate symptom management algorithms that generate guidance for a report delivered to clinicians in real-time. To our knowledge, this system is the first to provide CDS for management of multiple symptoms in oncology.

Another important facet of the SAMI-L system is that it produced immediate CDS for cancer symptom management based on complex logic utilizing patient data entered in real-time. The real-time collection of current symptom status from patients enabled SAMI-L to be responsive to the immediate needs of patients. The CDS tool was able to provide explicit advice for medication initiation or adjustment, as well as other interventions at the point-of-care. Enabling CDS to be responsive to current patient needs will become increasingly important as more data are collected in real-time through advances in patient-centric technologies.

From a technology standpoint, we validated the Web service approach for disassociating the collection of data and use of recommendations (in Massachusetts) from the decision engine (initially hosted on local servers in North Carolina and later moved to a cloud-based service). This project demonstrates that the client application can be separated from the decision engine over significant distances without compromising performance. The consistent function of SAMI-L demonstrates that Web service performance readily supports real-time, production-level-use CDS applications that deliver recommendations into workflow at the point-of-care. The Web service model would also accommodate potential reuse of the decision logic and scaling of the number of clients. As Dixon et al [45] note, provision of CDS by Web service opens the door to support for clinicians in settings with limited resources. Similar to the Dixon et al study, the SAMI-L decision engine could receive data from, and return decision support to, nonaffiliated health systems using secure protocols. Steurbaut et al [46] cite reduction of work overload as an additional advantage of a Web service approach.

This paper also demonstrates the magnitude of the testing required when implementing CDS using complex algorithms with over a hundred decision nodes and hundreds of possible values for the algorithm variables. The net result was more than a million possible unique data-parameter sets for traversing the most complex algorithm. The increased complexity of the logic supported by the SAMI-L CDS system necessitated new approaches to CDS testing. By using the STTS approach, we validated five complex CDS protocols for symptom management in cancer patients. In order to verify the accuracy of each algorithm, we automated the creation of hundreds of test data sets that enabled the assessment of boundary conditions, as well as the changing of multiple variables simultaneously. Thus, the STTS approach enabled boundary testing that would have otherwise been nearly impossible to achieve through a manual process, due to the protocol complexity. Moreover, this approach accommodated iterative testing of each protocol as it was refined by clinical experts, and allowed the testing process to be independent of the decision engine and the care protocol. In terms of generalizability, the testing framework used to validate SAMI-L can serve as a general model for testing CDS systems driven by complex algorithms in any clinical domain. In addition to the STTS approach, we manually constructed 10 sample cases derived from patients that reflected diverse symptomology, in order to test the entire system using all algorithms. We used this set of 10 test-cases to reassess system performance when modifications were made to the decision logic, since the change in the output reflects only the logic change, leaving all other recommendations constant.

One unanticipated issue was that hundreds of hours were needed to validate the algorithms before clinical implementation. In addition, a more iterative and user-centered design process between clinicians, research staff, and computer developers would have been ideal throughout the algorithm development cycle [47]. The expert panels produced algorithm flowcharts at the end of their work, and then programming of the decision rules began. When questions arose during programming, the expert panels were no longer meeting, and we had *ad hoc* access to only two clinical experts (palliative care and psychiatry), which created a slow and limited ability to address issues that arose during programming.

In terms of future directions for this work, SAMI-L should be tested in multiple clinics and used for symptom management for other types of cancer, especially in settings that have limited access to palliative care services [22]. In addition, the portability and shareability of the CDS logic via Web services should be demonstrated by allowing other CDS systems to use components of the SAMI-L knowledge base with new client applications.

### Limitations

One limitation of the current study is that we used paper copies of reports rather than integrating the system into the electronic health record (EHR) system. This approach was necessary, given the feasibility nature of the study, the need to establish efficacy of the technology, and the high cost of integrating the system within the EHR. It was important not to disrupt usual workflow, so our research staff worked collaboratively with the clinical staff to make sure the reports were readily accessible to HCPs prior to the clinic visits. Future studies that test the efficacy of this approach should explore mechanisms to integrate the technology into the EHR, ensuring that this approach has the potential to be broadly applied if efficacious.

### Conclusions

Complex algorithms can be invoked through rule-based CDS systems to promote evidence-based care in real-time at the point of patient contact using current, patient-supplied information to generate explicit, detailed, and patient-specific care guidance. This information collected in real-time from patients can be used to inform the symptom management process and serve to prioritize management interventions.

The increasing complexity of rule-based CDS systems requires new approaches to conduct thorough testing and validation of CDS systems, such as the STTS algorithm utilized in this project. Web services using a cloud-based decision engine can support clinical use of a CDS tool, in which the client application is independent and separate from the CDS engine.

## Appendix

Multimedia Appendix 1 Data requirements for the Symptom Assessment and Management Intervention (SAMI-L) System.

Multimedia Appendix 2 Symptom management guidance and required data elements for the algorithms.

## Abbreviations

BMC: Boston Medical Center
CDS: clinical decision support
DFCI: Dana-Farber Cancer Institute
EHR: electronic health record
HCC: Health Communication Core
HCP: health care provider
SAMI-L: Symptom Assessment and Management Intervention for Lung cancer
SEBASTIAN: System for Evidence-Based Advice Through Simultaneous Transaction with an Intelligent Agent Across a Network
STTS: stack-traversal tree-spanning
XML: eXtensible Markup Language
