# Supplementary material for: Increasing Complexity in Rule-Based Clinical Decision Support: The Symptom Assessment and Management Intervention
Source: JMIR Med Inform. 2016 Nov 8;4(4):e36. doi: 10.2196/medinform.5728 (PMC5120240; doi:10.2196/medinform.5728)
Supplement: Multimedia Appendix 1 [file medinform_v4i4e36_app1.pdf]

Multimedia Appendix 1. Data requirements for the Symptom Assessment and Management Intervention (SAMI-L) System.

Domain	Required Data Elements	Source of Data	Instrument / Data Collection Method
Symptom Characteristics	Pain severity (mild, moderate, severe)	Patient	Pain self-assessment numerical rating scale and single items describing quality of pain
	Pain sensation (neuropathic, somatic, or both)		
	Pain pattern (constant, intermittent)		
	Insomnia severity		Insomnia self-assessment, single item, symptom distress scale (SDS)[24]
	Anorexia severity		Appetite self-assessment, single item, SDS
	Nausea frequency and severity		Nausea self-assessment, two items, SDS
	Dyspnea severity<br>(mild, moderate, severe)		Dyspnea self-assessment, single item, SDS
	Severity of fatigue<br>(mild, moderate, severe)		Fatigue self-assessment, single item, SDS
	Bowel function/ constipation severity		Bowel self-assessment, single item, Patient Reported Outcomes version of the Common Terminology Criteria for Adverse Events (PRO-CTCAE)[25]
	Depression severity<br>(Normal, mild, moderate, severe)		Depression self-assessment, 9 items, Patient Health Questionnaire (PHQ-9) patient health questionnaire - 9 item[26]
	Anxiety severity<br>(normal, mild, moderate, severe)		Anxiety self-assessment, 7 items, Hospital Anxiety and Depression Scale (HADS), anxiety subscale[27]
Clinical Parameters	Age	Research	Obtained from measure of vital signs

	Weight	Staff	and laboratory test results at each visit
	Creatinine		
	Platelets		
	Hemoglobin		
Comorbidities/ Past Medical History	History of peptic ulcer disease	Patient	Verbally conveyed to research staff by patients or medical records
	Alcohol use		Alcohol Use Disorder Inventory Test (AUDIT): Hazardous Alcohol Use subscale, 1-3 items-- results used to identify hazardous drinking in the face of prescribing opioids and sedating medications[37]
	Previous report of depression		Obtained from patient self-report on PHQ-9
	Anhedonia		
	Suicidal ideation		
	Previous report of anxiety		Obtained from patient self-report on HADS
	Previous report of fatigue		Obtained from patient self-report on SDS
Relevant Medication Details	Supportive care medications prescribed	Research Staff	Medical record prescriptions for medications for pain, dyspnea, depression, anxiety and fatigue
	Supportive care medications start date, dose, and frequency taken		
	Supportive care medications reasons for non-adherence		
