# Supplementary material for: Increasing Complexity in Rule-Based Clinical Decision Support: The Symptom Assessment and Management Intervention
Source: JMIR Med Inform. 2016 Nov 8;4(4):e36. doi: 10.2196/medinform.5728 (PMC5120240; doi:10.2196/medinform.5728)
Supplement: Multimedia Appendix 2 [file medinform_v4i4e36_app2.pdf]

Multimedia Appendix 2. Symptom management guidance and required data elements for the algorithms.

Symptom	Pharmacologic and Behavioral Management Considerations	Required Data Elements
Pain	• Calculates opioid adjustment doses based on severity of pain, previous use of opioids, amount of medication required over the past 24 hours, and renal function<br>• Calculates conversions for preferred opioids<br>• Starts treatment with adjuvant medications for neuropathic and/or somatic pain, taking into consideration the presence of co-existing platelet count, renal function, and history of peptic ulcer disease<br>• Initiates or up titrates a bowel regimen to prevent or treat opioid-induced constipation	• Pain severity (mild, moderate or severe)<br>• Age<br>• Platelet count<br>• Renal function (creatinine clearance calculation)<br>• History of peptic ulcer disease<br>• Bowel function/constipation severity<br>• Bowel medication use<br>• Pain sensation (neuropathic, somatic, or both)<br>• Pain pattern (constant, intermittent<br>• Current opioid use (opioid naïve, or current opioid strength and dosage forms)
Dyspnea	• Calculates opioid adjustment doses based on severity of dyspnea, previous use of opioids and renal function;<br>• Calculates conversions for preferred opioids<br>• Recommends treatment for anemia<br>• Initiates or up-titrates a bowel regimen to prevent or treat opioid-induced constipation	• Dyspnea severity (3 levels),<br>• Hemoglobin level<br>• Renal function (creatinine clearance calculation)<br>• History of peptic ulcer disease<br>• Current opioid use (opioid naive, or opioid strengths and dosage forms)<br>• Pain severity<br>• Bowel function/constipation severity<br>• Bowel medication use<br>• Pain sensation (neuropathic, somatic)<br>• Age<br>• Platelet count

Depression	• Recommends treatment for depression based on level of severity and suggests continuing or up-titrating medications depending on response<br>• Medications are tailored based on presence of nausea, insomnia and/or anorexia<br>• Provides cross-taper of medication choice for refractory depression<br>• Suggests laboratory tests and referral to psychiatry for further evaluation for patients with refractory depression<br>• Recommends referral to a social worker for mild, or psychiatrist for refractory, depression<br>• Flags suicidal ideation	• Depression severity (4 levels)<br>• Previous report of depression<br>• Anhedonia<br>• Suicidal ideation<br>• Pain severity<br>• Fatigue severity<br>• Insomnia severity<br>• Anorexia severity<br>• Nausea frequency and severity<br>• Medication dosing and start date of 3 medication classes
Anxiety	• Recommends treatment for anxiety based on frequency of anxiety and level of severity and suggests continuing or up-titrating medications depending on response<br>• Medications are tailored based on presence of nausea, insomnia and/or anorexia<br>• Provides cross-taper of medication choice for refractory anxiety<br>• Suggests laboratory tests and referral to psychiatry for further evaluation when patients fail long-term anxiolytic use	• Anxiety severity (4 levels)<br>• Previous report of anxiety<br>• Pain severity<br>• Dyspnea severity<br>• Insomnia severity<br>• Anorexia severity<br>• Nausea frequency and severity<br>• Medication dosing and start date of 3 medication classes
Fatigue	• Recommends laboratory testing and correction of anemia<br>• Suggests gradual discontinuation of benzodiazepines unless needed for anxiety<br>• Suggests sleep medication at night for patients with insomnia<br>• Recommends stimulant medication +/- physical therapy consult based on severity of fatigue and anemia<br>• Recommends referral to a social worker for mild, or psychiatrist for refractory, anxiety	• Severity of fatigue (3 levels)<br>• Previous report of fatigue<br>• Pain severity<br>• Dyspnea severity<br>• Depression severity<br>• Anhedonia<br>• Benzodiazepine use<br>• Insomnia severity<br>• Hemoglobin level
